# Prognostic and immunotherapeutic significance of immunogenic cell death-related genes in colon adenocarcinoma patients

**DOI:** 10.1038/s41598-023-46675-y

**Published:** 2023-11-06

**Authors:** Jun Xu, Jun Yang, Xianzhu Pan, Jian Wang

**Affiliations:** 1 Department of Basic Courses, Anhui Medical College, Hefei, 230032 Anhui China; 2 Department of Pathology, Hefei First People’s Hospital, Hefei, 230001 Anhui China

**Keywords:** Data mining, Gene ontology, Cancer, Computational biology and bioinformatics

## Abstract

In recent years, genes associated with immunogenic cell death (ICD)-related genes have garnered significant interest as potential targets for immunotherapy. As a frontier in cancer treatment, immunotherapy has notably enhanced the therapeutic outcomes for cancer patients. However, since only a subset of patients benefits from this treatment approach, there is an imperative need for biomarker research to enhance patient sensitivity to immunotherapy. Expression of ICD-related genes and clinical patient data were sourced from The Cancer Genome Atlas (TCGA) database. Utilizing univariate Cox regression analysis, we constructed a signature for predicting the overall survival of colon adenocarcinoma (COAD) patients. A genomic feature analysis was performed, incorporating tumor mutation burden (TMB) and copy number variation (CNV). The immunological characteristics were analyzed via the ssGSEA and GSEA algorithms, with the resulting data visualized using R software (version 4.2.1). According to the univariate regression analysis for COAD, AIM2 emerged as the gene most significantly associated with overall survival among the 32 ICD-related genes in the TCGA dataset. Patients were divided into two groups based on high or low AIM2 expression, and genomic differences between the groups were explored. Patients expressing high levels of AIM2 had a higher TMB and a lower CNV. In addition, these patients had elevated immune checkpoint, immune cell, and immune function scores, thus indicating increased sensitivity to immunotherapy. TIDE analysis further confirmed that these patients were likely to respond more effectively to immunotherapy. Subclass mapping analysis corroborated our findings, demonstrating that patients with high AIM2 expression responded more positively to immunotherapy. Additionally, our study found that the suppression of AIM2 could significantly enhance the proliferation, invasion, and migration capabilities of colon cancer cells. In this research, we identified a novel prognostic signature suggesting that patients with higher AIM2 expression levels are more likely to respond favorably to immunotherapy.

## Introduction

Colon adenocarcinoma (COAD) is a highly prevalent form of human malignant tumors, with approximately 1,148,000 new cases and 576,000 new deaths reported globally in 2020^1^. The projections for 2030 predict about 2.2 million new cases and 1.1 million fatalities^2^. Presently, surgery remains the primary treatment for early-stage COAD patients. However, most COAD patients are diagnosed at advanced stages and metastatic recurrences are commonly observed even after the primary tumor has been removed^3^. Despite substantial advancements in combined therapy strategies for COAD, patients often face low 5-year survival rates^4^. While the discovery of new biomarkers in COAD has enhanced diagnostic and therapeutic strategies, the heterogeneity of COAD undermines the efficacy of single biomarkers^5^. Consequently, identifying more precise biomarkers is crucial for the development of individualized prognosis and treatment plans for COAD patients.

Research suggests that cancer cells modify the tumor microenvironment to influence immune cells, leading to immune evasion and cancer progression^6^. Interestingly, studies have identified that external stimuli can cause tumor cells to die and become immunogenic, leading the immune system to trigger an anti-tumor response—a process known as immunogenic cell death (ICD)^7^. During ICD, various signaling molecules are released by the tumor cells, initiating a series of cytological reactions that ultimately activate the innate and adaptive immune systems^8^. By understanding how tumor cells induce ICD, we can stimulate ICD in cancer cells, dismantle the inhibitory tumor microenvironment, and reestablish anti-tumor effects. To date, research on COAD and ICD has been sparse. Thus, it is crucial to explore COAD’s bio-information and prognostic capabilities to identify new biomarkers and therapeutic targets.

In this study, we conducted an integrated analysis of the characteristics of ICD-related genes to evaluate the impact of the expression of these genes on the survival, prognosis, and response to immunotherapy in COAD patients, especially AIM2. We hoped this study would enhance the diagnosis and prognosis for COAD.

## Methods

### Data collection and processing

Normalized transcriptome profile data of 514 samples (473 COAD samples and 41 normal samples) were downloaded from The Cancer Genome Atlas (TCGA) database (https://tcga-data.nci.nih.gov/tcga)^9^. The gene expression data of additional normal tissue were also accessed from the Genotype-Tissue Expression (GTEx) database (https://gtexportal.org/)^10^. The data were then processed in the following manner: (1) The genome reference file was employed for probe annotation; (2) The expression matrix was merged and preprocessed; (3) The clinical data and gene expression data from all COAD samples were combined, and patient data with survival times less than 1 month were excluded to mitigate the influence of analytical errors.

### Differing expression analysis

Initially, 32 ICD-related genes were derived from the literature^11^. Subsequently, data for 29 ICD-related genes in COAD were retrieved from the TCGA and GTEx databases and visualized utilizing the “limma” and “ggplot2” R packages^12^.

### Assess the prognostic characteristics of COAD patients

Excluding patients with a survival time of less than a month, univariate Cox regression analysis was conducted on 432 COAD patients from the TCGA database, to determine which ICD-related genes were associated with prognosis. Based on the expression of the selected genes, the optimal truncation value was determined and patients were divided into high and low expression groups. Pie charts were used to depict the distribution of clinical information across these two groups. Furthermore, we compared the AIM2 gene expression in different M stages and clinical stages (stages I–II or stages II–IV) to ascertain whether it varied between these groups of patients.

### Analyzing tumor mutation burden

Considering the high incidence of gene mutations in COAD patients and the prominence of mutated genes, the tumor mutational burden (TMB) was assessed using somatic mutation data acquired from the cBioPortal website (https://www.cbioportal.org/datasets)^13^. We compared TMB levels between the two groups and identified the genes with the most mutations, which might be COAD-driving genes. The TMB levels in COAD could be better visualized using pan-cancer TMB data from cBioPortal, which facilitates comparison with other cancer types. In addition, we compared the prognosis of patients with high and low TMB. An upsurge in the expression of ICD-related genes can instigate ICD in tumor cells. Subsequently, KM survival analysis was deployed to compare survival times of patients from high and low mutation groups in correlation with high and low expression groups.

### Comparison of stemness index and copy number variation

Tumor stem cells expedite tumor progression, metastasis, drug resistance, and self-renewal through self-protective mechanisms such as DNA damage repair. The mRNA expression-based stemness index (mRNAsi) and the DNA methylation-based stemness index (mDNAsi) were computed using the OCLR algorithm^14^. We also conducted a differential analysis of the tumor stemness index between the two groups. Furthermore, we analyzed copy number variation (CNV) between two groups using the online application GISTIC 2.0 (https://cloud.genepattern.org). To identify any differences in gene amplification or deletion between the two groups, a differential analysis was performed. In addition, the burden of copy number loss and gain between the two groups was analyzed both at the arm and focal levels.

### Gene set enrichment analysis of differentially expressed genes (DEGs)

We employed the “limma” package of R software to filter DEGs between the two groups, using selection parameters such as P < 0.05 and a fold-change > 2 or fold-change < − 1. The study encompassed all kinds of gene sets, including C1 to C8 and hallmark gene sets obtained from the MSigDB Collections (https://www.gsea-msigdb.org/gsea/msigdb/collections.jsp). The Gene Set Enrichment Analysis (GSEA) was deployed to inspect the overall direction of DEGs enrichment in these pathways^15^. For patients from both groups, we conducted a pathway enrichment analysis using Gene Ontology (GO) and the Kyoto Encyclopedia of Genes and Genomes (KEGG), with the aim of interpreting the biological significance of DEGs^16–18^. DEGs were enriched and analyzed in hallmark gene sets using GSEA, and then visualized with ggplot2^19^.

### Immune terms, immune infiltration and immunotherapy

Single Sample Gene Set Enrichment Analysis (ssGSEA) was performed to probe the connection between AIM2 expression and 13 immune-related function scores in COAD patients^20^. CIBERSORT algorithm was used to quantify the immune infiltration microenvironment of colon cancer tissue^21^. Leveraging the broad use of Immune Checkpoint Inhibitors (ICIs) in cancer treatment, we also studied the relationship between AIM2 gene expression and the expression of four prevalent immune checkpoints—PD-1, PD-L1, PD-L2, and CTLA4. We executed a comparative analysis of the expression of these four immune checkpoints between the two groups, and also compared the expression of 50 immune checkpoints. Utilizing the ssGSEA algorithm, we analysed and graphically represented the variations in the expression of immune cells and immune-related function scores. Next, we computed immune scores, stromal scores, and ESTIMATE scores with the help of the “estimate” R package. Guided by the differential results in immune infiltration between the two groups, we performed a comparative analysis of immunotherapy responses using Tumor Immune Dysfunction and Exclusion (TIDE) analysis^22^. Subsequently, subclass mapping algorithms were employed to evaluate PD-1 and CTLA4 treatment effects on patients in the two groups. Lastly, we used the ‘oncoPredict’ R package to ascertain drug sensitivity, including the half maximal inhibitory concentration (IC50), and to predict potential targeted therapy drugs.

### Cell culture and transfection

The cell line SW480 along with human monocytes cell line THP-1, were all obtained from the American Type Culture Collection (ATCC, Manassas, VA), whchi were cultured under the condition of 37 °C and 5% CO_2_. Cells are subcultured every 3–4 days. Cell transfection was performed using Lipofectamine 2000, according to the manufacturer's instructions. Detailed, SW480 cells were seeded into culture plates and allowed to reach a suitable confluence (80%) for transfection. Meanwhile, the transfection complexes were prepared. For each well, an appropriate amount of shRNA was diluted in Opti-MEM. In a separate tube, Lipofectamine 2000 was similarly diluted in the same transfection medium. The Lipofectamine solution was gently mixed and then incubated at room temperature for 10 min. After incubation, the diluted shRNA and Lipofectamine solutions were combined and gently mixed. The resulting transfection complexes were allowed to form during a further incubation of 20 min. The culture medium from the SW480 cells was aspirated, and the cells were gently washed with phosphate-buffered saline (PBS). The transfection complexes were then added to the cells, ensuring even coverage. The cells were incubated with the transfection complexes at 37 °C with 5% CO_2_ for an appropriate duration (6 h). Following the incubation, the transfection medium was replaced with fresh growth medium, and the cells were allowed to recover. The shRNAs were purchased from Genomeditech (Shanghai, China). The target sequences were: shRNA#1: GTGGTTTCTTAGAGGTAAA; shRNA#2: GGAGTTCATAGCACCATAA; shRNA#3: GTCCCGCTGAACATTATCA.

### Quantitive real-time PCR (qPCR)

Total RNA was extracted using an RNA simple Total RNA extraction kit and then was reversely transcribed into cDNA. SYBR Green system was applied to perform qPCR. SYBR Green qPCR reactions were set up in a 96-well plate. Each reaction consisted of cDNA, gene-specific primers, and SYBR Green Master Mix (20 μL system). The obtained data were analyzed using qPCR analysis software. The cycle threshold (Ct) values were determined for each gene of interest and reference gene. The relative gene expression levels were calculated using the comparative Ct (2^−ΔΔCt^) method or other suitable algorithms. The primers used were: AIM2, forward, 5′-TGGCAAAACGTCTTCAGGAGG-3′, reverse, 5′-AGCTTGACTTAGTGGCTTTGG-3′; GAPDH, forward, 5′-GGAGCGAGATCCCTCCAAAAT-3′, reverse, 5′-GGCTGTTGTCATACTTCTCATGG-3′.

### Tumor formation experiment in nude mice

We carefully selected and maintained male BALB/c nude mice, aged between 4–6 weeks, under stringent sterile conditions throughout the duration of the study. To establish the in vivo model, we prepared a human tumor cell suspension containing 1 × 10^6^ cells, which was subsequently diluted with PBS (shRNA#2 and control group). Each mouse received a subcutaneous inoculation of 0.1 mL of the cell suspension under the right forelimb. After 6 weeks of the nude mouse tumor experiment, all nude mice were euthanized. The tumors were excised from the mice for further evaluation as part of our experimental protocol. Tumor tissue sections underwent immunohistochemical staining for Ki67, a well-established cell proliferation marker. To ensure the highest quality of results, the following steps were meticulously followed: The tumor tissue sections were initially heated in a 60 °C oven for a two-hour period to enhance tissue preparation. Subsequently, the sections underwent antigen unmasking through microwave heating in citrate buffer (pH 6.0). To block endogenous peroxidase activity, the sections were immersed in a solution of 3% ketone hydroperoxide in methanol for a 20-min duration at room temperature. To prevent non-specific binding, the sections were then treated with PBS enriched with 10% normal goat serum. Following this, the Ki67 antibody (diluted at 1:200 in blocking solution) was meticulously applied to the sections and allowed to incubate overnight at 4 °C. The next day, the sections were coated with biotinylated secondary antibodies, followed by a further hour-long incubation. A chromogenic substance, DAB, was subsequently applied to visualize the staining. To provide contrast and enhance visualization, hematoxylin–eosin staining was performed. Finally, the stained sections were examined under a microscope to accurately calculate the percentage of cells that tested positive for Ki67.

### Lung metastasis model

In the lung metastasis model, we employed male BALB/c nude mice aged between 4–6 weeks. To initiate the experiment, we meticulously prepared a suspension of human tumor cells, consisting of 1 × 10^6^ cells, which was subsequently diluted to a volume of 200 μL using PBS (shRNA#2 and control group). This cell suspension was administered to the mice through tail vein injection. After 2 weeks from the commencement of the experiment, we diligently conducted observations of the mice’s survival, which continued for a total duration of 60 days. Upon the conclusion of this observation period, we humanely euthanized the mice, and their lung tissues were then carefully excised and subjected to thorough assessment for the presence of microscopic metastatic nodules, employing the hematoxylin–eosin (H&E) staining technique.

### Co‐culturing system

In order to emulate the interaction between tumor cells and tumor-associated macrophages (TAMs), we established an indirect co-culture model in vitro. We seeded macrophages and colon cancer cells separately into the upper and lower chambers of a Corning^®^ Transwell^®^ cell culture insert (4 μm pore size, Corning Inc., Corning, NY, USA), which featured a polycarbonate membrane. Following a co-culture period of 48 h, we collected the cells for additional experiments.

### Macrophage induction from monocytes and flow cytometry

We cultured THP-1 cells in six-well plates, and treated them with 100 ng/mL of phorbol-12-myristate-13-acetate (PMA; Sigma-Aldrich) for a period of 24 to 48 h, thus transforming them to THP-1 (Mφ). After incubation, we replaced the medium with fresh PMA-free medium, allowing the cells to grow for another 3 days before utilization. For identifying macrophage surface markers, we exposed cells suspended in cold PBS to either anti‐CD206 or anti‐CD86 antibodies (sourced from eBioscience), maintaining the mixtures at 4 °C for half an hour. After incubation, we washed the cells and employed a BD Accuri™ C6 flow cytometer (BD Biosciences, San Jose, CA, USA) for the analysis of macrophage surface markers CD206 and CD86.

### Cell proliferation assay

Cell proliferation ability was evaluated using the CCK8 and colony formation assay, which were performed as previously described^23^. In detail, for CCK8 assay, cells were trypsinized and counted. A predetermined number of cells (1000 cells per well) was then seeded into a 96-well plate containing the appropriate culture medium. Use multiple wells per condition or time point. Cells were cultured under standard conditions, typically at 37 °C in a humidified atmosphere containing 5% CO_2_. After the designated incubation period, aspirate the culture medium and add CCK-8 solution to each well. Prepare solutions according to manufacturer's instructions and add to cells. The plate was then returned to the incubator for the specified time to allow the CCK-8 reagent to be metabolized by viable cells. After incubation, measure the optical density (OD) of each well at a specific wavelength (450 nm) using a microplate reader. For colony formation assay, a predetermined number of cells (500 cells per well) were seeded into 6 wells. Care is taken to ensure that each cell is separated from other cells to form a separate colony (2 weeks). The cells are then fixed, usually with methanol, and stained crystal violet, and the colonies are counted using a light microscope.

### Transwell assay

Transwell assay was used to evaluate the invasion and migration ability of colon cancer cells, which was performed as previously described^23^. For Transwell assays, cells were serum-starved overnight to synchronize the culture. Transwell inserts were placed in a 24-well plate. Matrigel-coated inserts were used for invasion assays, while uncoated inserts were used for migration. Serum-free medium was added to the upper chamber, and cells were resuspended in a minimal volume of serum-free medium. The cell suspension (1 × 10^6^ cells) was added to the upper chamber. For migration and invasion assays, serum-containing medium was added to the lower chamber. The plate was then incubated at 37 °C in a 5% CO_2_ incubator, allowing cells to migrate or invade for a specified time (24 h). After incubation, non-migrated/invaded cells on the upper side of the membrane were removed. The migrated/invaded cells on the lower side of the membrane were fixed, stained, and counted or imaged for analysis.

### Statistical analysis

R, GraphPad Prism 8 and SPSS software were used for all statistical analysis. The statistical threshold was set as 0.05. Different statistical methods were utilized based on different data distribution forms. The experiments were repeated three times.

## Results

### Data combination

**Figure 1:**
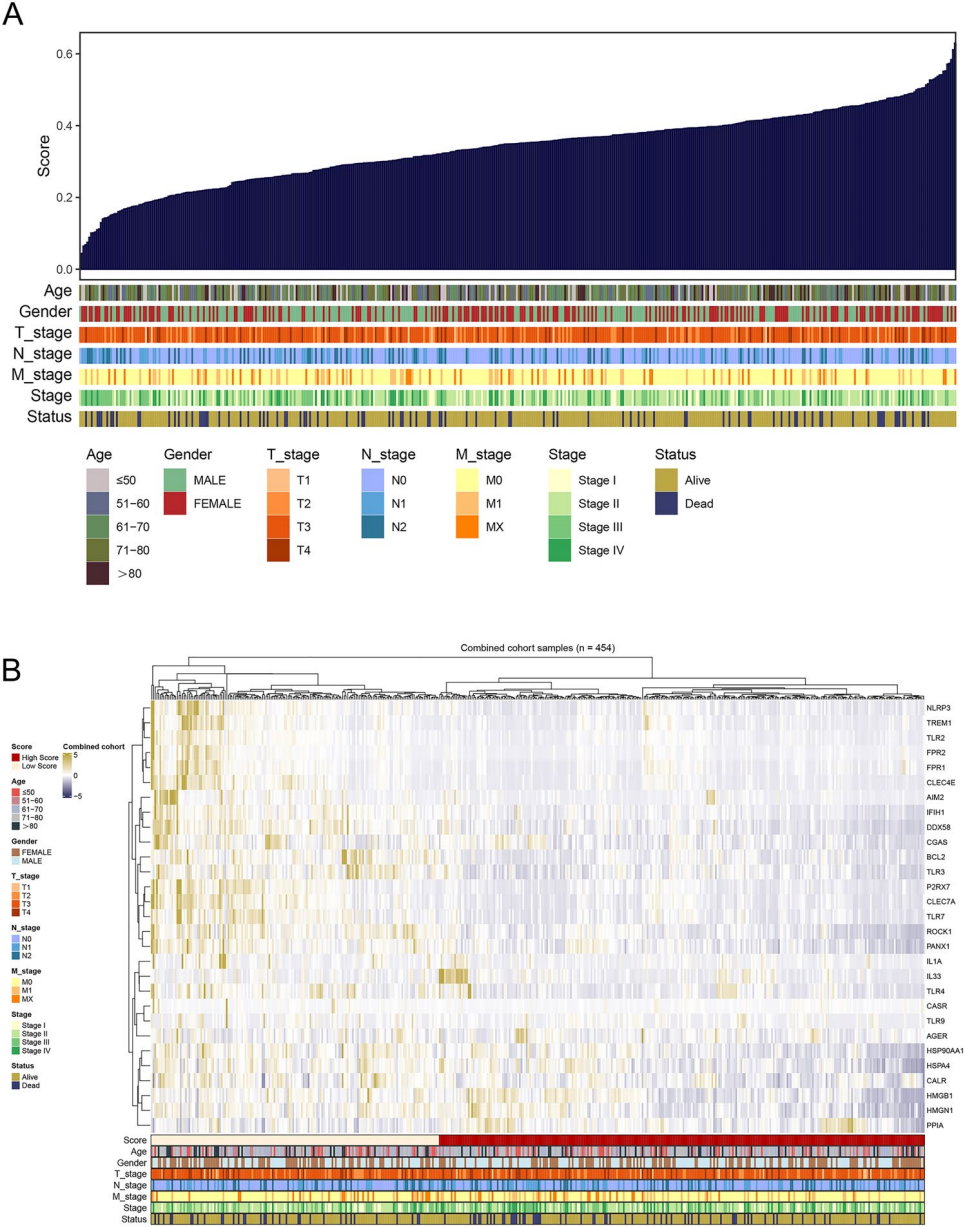
Identification of immune scores and the expression of ICD-related genes in TCGA cohort. (**A**) The association of immune scores and clinical characteristics (age, gender, T stage, N stage, M stage, stage and survival status) in COAD. (**B**) Heatmap showing the expression of 29 immune-related genes.

Utilizing the ssGSEA algorithm, we computed the scores for each patient's ICD-related genes based on the TCGA-COAD dataset (Fig. 1A). Upon evaluating the results from COAD patients, we observed a heatmap that displayed both the expression of ICD-related genes and the corresponding clinical information characteristics (Fig. 1B).

### ICD-related genes-based prognosis signature

**Figure 2:**
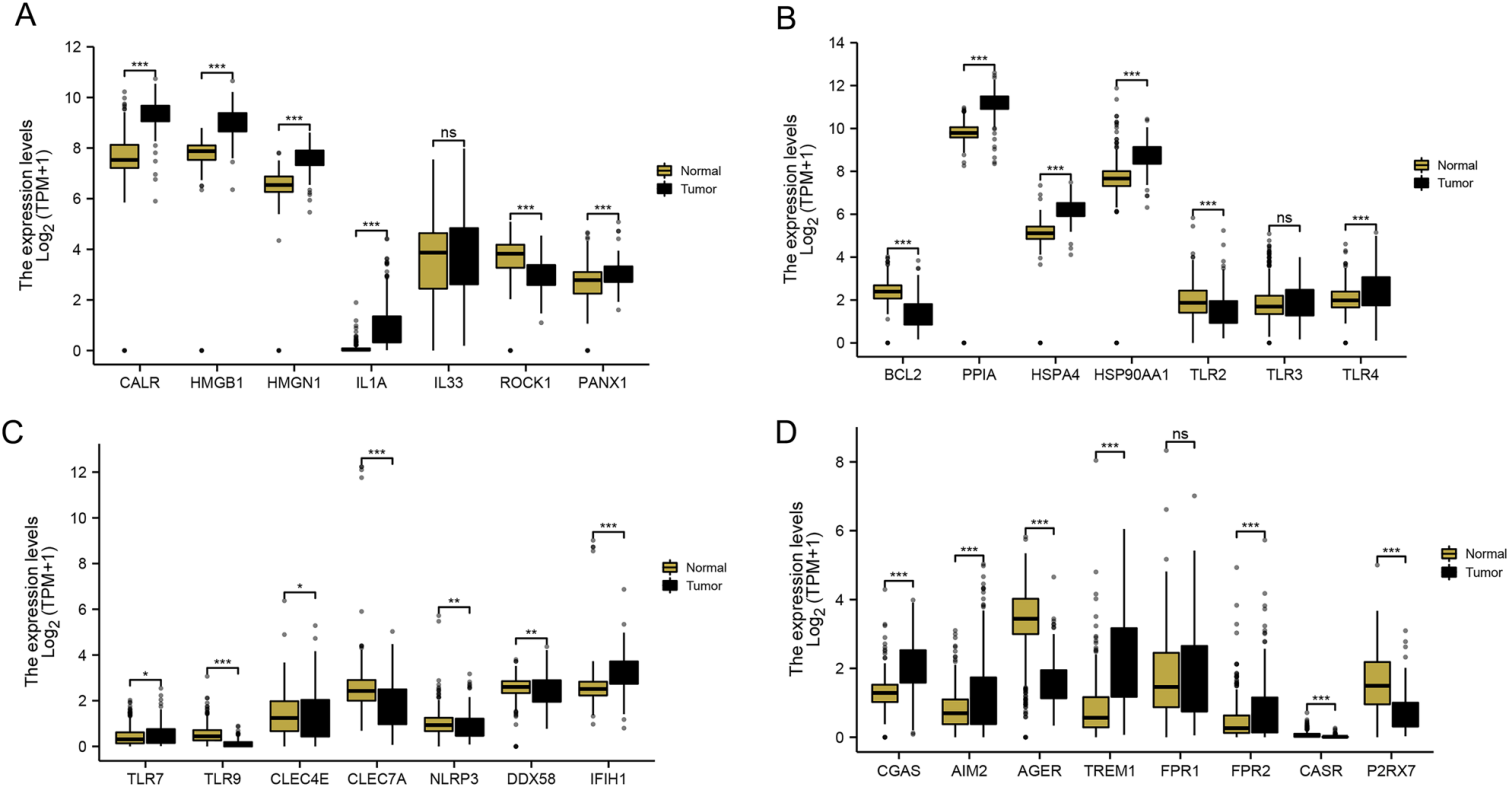
The expression of ICD-related genes in COAD. (**A**–**D**) Differential expression analysis of expression of ICD-related genes between tumor and normal samples, ^ns^P > 0.05, *P < 0.05, **P < 0.01, ***P < 0.001 (independent samples *t* test).

**Figure 3:**
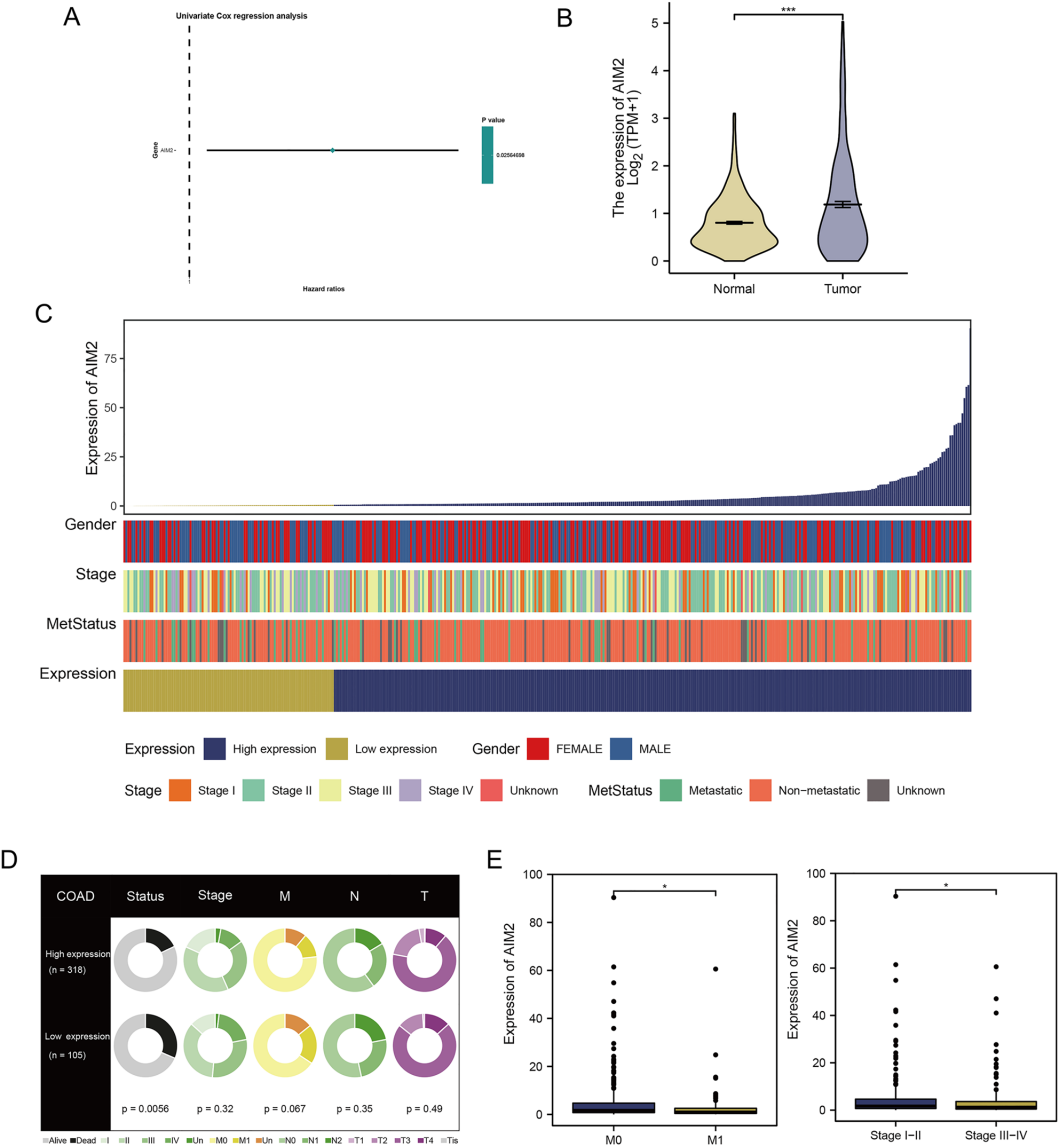
Identification of ICD-related prognostic signature. (**A**) Univariate Cox regression analysis screened 1 ICD-related gene. (**B**) The comparison of AIM2 expression between tumor and normal samples, ***P < 0.001 (independent samples *t* test). (**C**) Clinical features associated with the expression of AIM2. (**D**) Distribution of clinical features between high and low expression groups. (**E**) The comparison of AIM2 expression in different M stage and clinical stage, *P < 0.05 (independent samples *t* test).

Antitumor responses can be induced by ICD-related genes. By juxtaposing tumor and normal samples from TCGAs and GTEx, we were able to identify significant disparities in the expression of ICD-related genes (Fig. 2A–D). Out of 21 ICD-related genes with notably different expressions, seven genes, namely ROCK1, BCL2, TLR2, TLR9, AGER, CASR, and P2RX7, were found to have decreased expression in tumor samples, while the rest were upregulated (Fig. 2A–D). Additionally, we employed univariate analysis to pinpoint prognosis-associated genes. Patients who survived for more than 30 days were the primary subjects of further analysis, amounting to a total of 432 patients. The univariate Cox regression analysis identified AIM2 as the gene of particular interest (Fig. 3A). We found that tumor samples expressed higher levels of AIM2 compared to normal samples (Fig. 3B). In particular, we explored the impact of AIM2 on tumor cell apoptosis and proliferation. With patients categorized into high and low expression groups based on an optimal truncation value determined by AIM2 gene expression (Fig. 3C), we discovered distinct variations in survival status, clinical stage, M stage, N stage, and T stage between the two groups, suggesting that the patients in the low expression group faced more serious conditions than their counterparts in the high expression group (Fig. 3D). It was also evident that the AIM2 gene expression varied among patients in the M0 and M1 stage, as well as those in stages I–II and stages II–IV (Fig. 3E).

### Mutation analysis

**Figure 4:**
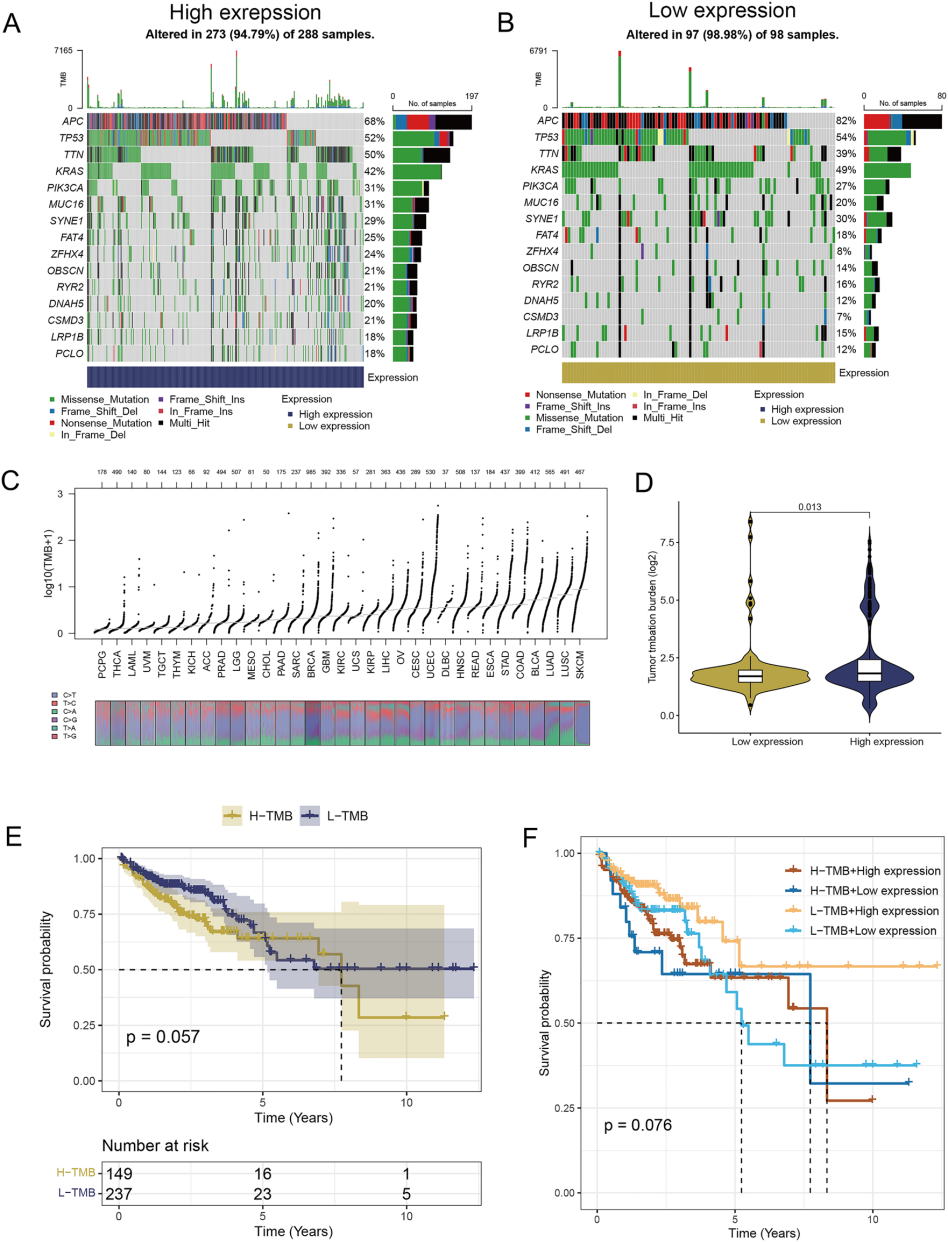
Identification of TMB levels between high and low expression groups. (**A**,**B**) Mutation profiles of high and low expression groups. (**C**) Distribution of TMB levels in pan-cancer. (**D**) Comparison of TMB levels between two groups (independent samples *t* test). (**E**) Kaplan–Meier plot for overall survival based on the TMB levels of patients (Log-rank test). (**F**) Survival curve of patients based on the TMB levels and different expression groups (Log-rank test).

We evaluated the TMB in both high and low expression groups to ascertain whether AIM2 expression was associated with TMB levels. Notably, COAD featured specific genes with mutations, with APC, TP53, TTN, and KRAS being the most commonly mutated genes in both high and low expression groups (Fig. 4A,B). TMB levels across all cancer types were determined from the TCGA database, and it was observed that COAD had a higher level of TMB compared to other types of cancers (Fig. 4C). Generally, patients with high TMB levels tend to have better immunotherapy outcomes as T cells activated by ICIs can more easily recognize and attack neoantigens in tumor cells^24^. In our study, patients in the high expression group exhibited significantly elevated TMB levels compared to those in the low expression group (Fig. 4D). Consequently, patients with higher AIM2 expression had a higher TMB and more neoantigens, thereby improving the likelihood of tumor cells being eliminated by T cells. With elevated TMB and AIM2 expressions, tumors were more prone to immunogenic cell death, potentially benefiting the patients undergoing immunotherapy. However, when comparing the overall survival (OS) times between high and low mutation groups, the survival trends were not significantly different (Fig. 4E). Patients with higher expression of ICD-related genes might benefit from immune checkpoint blockers. Notably, a prolonged survival rate was observed among patients with low TMB and high AIM2 expression (Fig. 4F).

### Stemness index calculation and copy number variation

**Figure 5:**
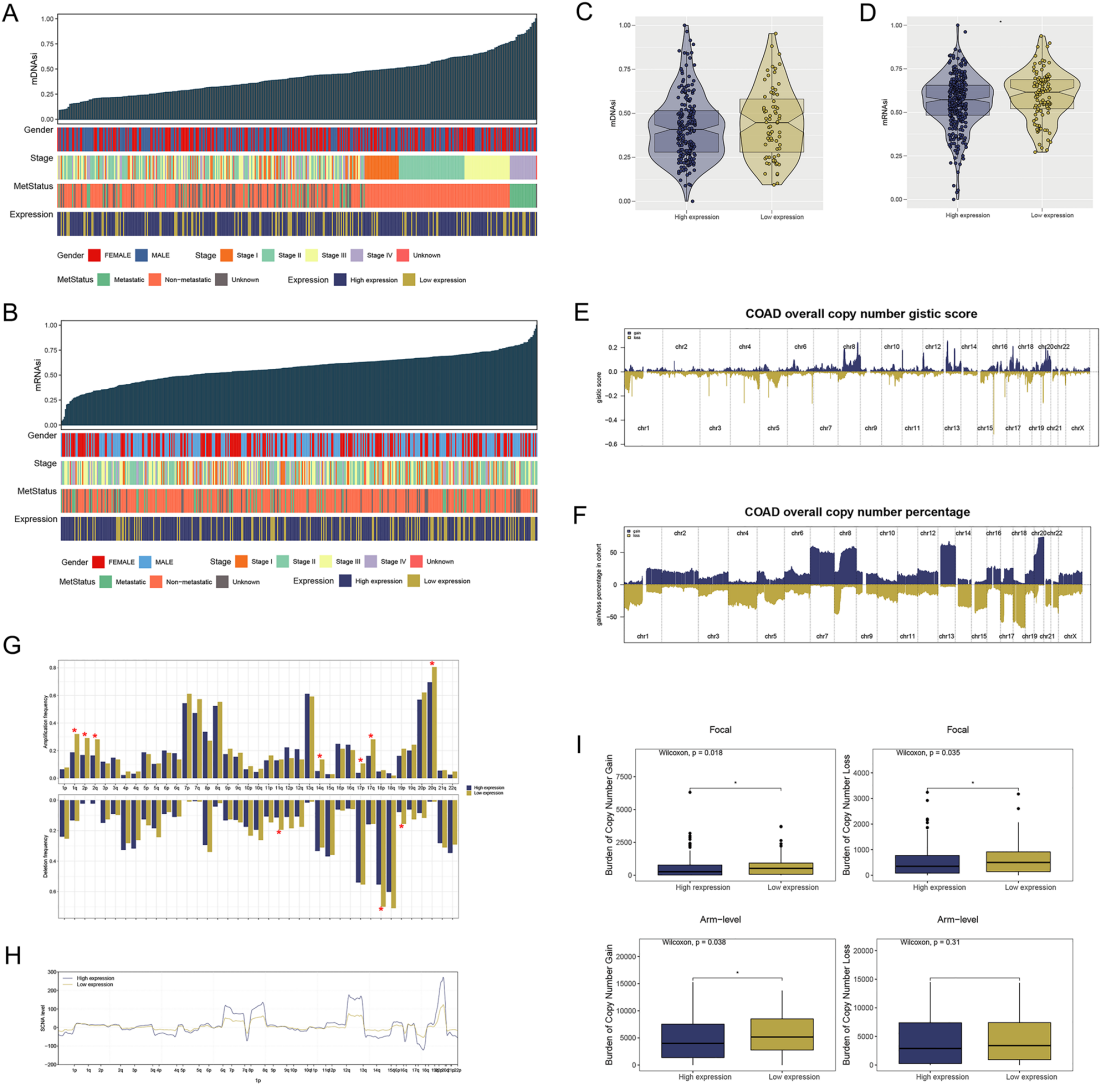
Analyses of mDNAsi and mRNAsi in patients. (**A**,**B**) The association between clinical characteristics (gender, stage, metastasis and expression groups) and mDNAsi as well as mRNAsi in COAD. (**C**,**D**) The comparison of mDNAsi and mRNAsi between two expression groups, *P < 0.05 (independent samples *t* test). (**E**,**F**) Copy number burdens of gain and loss of genes for two expression groups. (**G**,**H**) The comparison of amplification and deletion frequencies of chromosomes for two expression groups (Mann–Whitney *U* test). (**I**) Comparison of copy number gain and loss based on focal and arm levels between two expression groups, *P < 0.05 (independent samples *t* test).

Tumor stem cells play a crucial role in tumor growth, proliferation, metastasis, and recurrence^25^. Therefore, we illustrated the distributions of mDNAsi and mRNAsi in conjunction with clinical information (Fig. 5A,B). Both mDNAsi and mRNAsi were lower in the high expression group than the low expression group, suggesting an increased susceptibility of high expression group tumors to ICD (Fig. 5C,D). Moreover, we computed gistic scores and percentages for each COAD patient to explore the CNV differences between the two groups (Fig. 5E,F). The frequencies of amplification and deletion between high and low expression groups were compared and the level of SCNA was illustrated with a line diagram (Fig. 5G,H). In the low expression group, the amplification frequencies of 1q, 2p, 2q, 14q, 17p, 17q, and 20q were statistically higher than those in high expression group, as well as the deletion frequencies of 11q, 18p, and 19p, respectively. The differential CNV analysis indicated a higher prevalence of focal and arm CNV loss or gain in the low expression group compared to the high expression group (Fig. 5I).

### Gene set enrichment analyses

**Figure 6:**
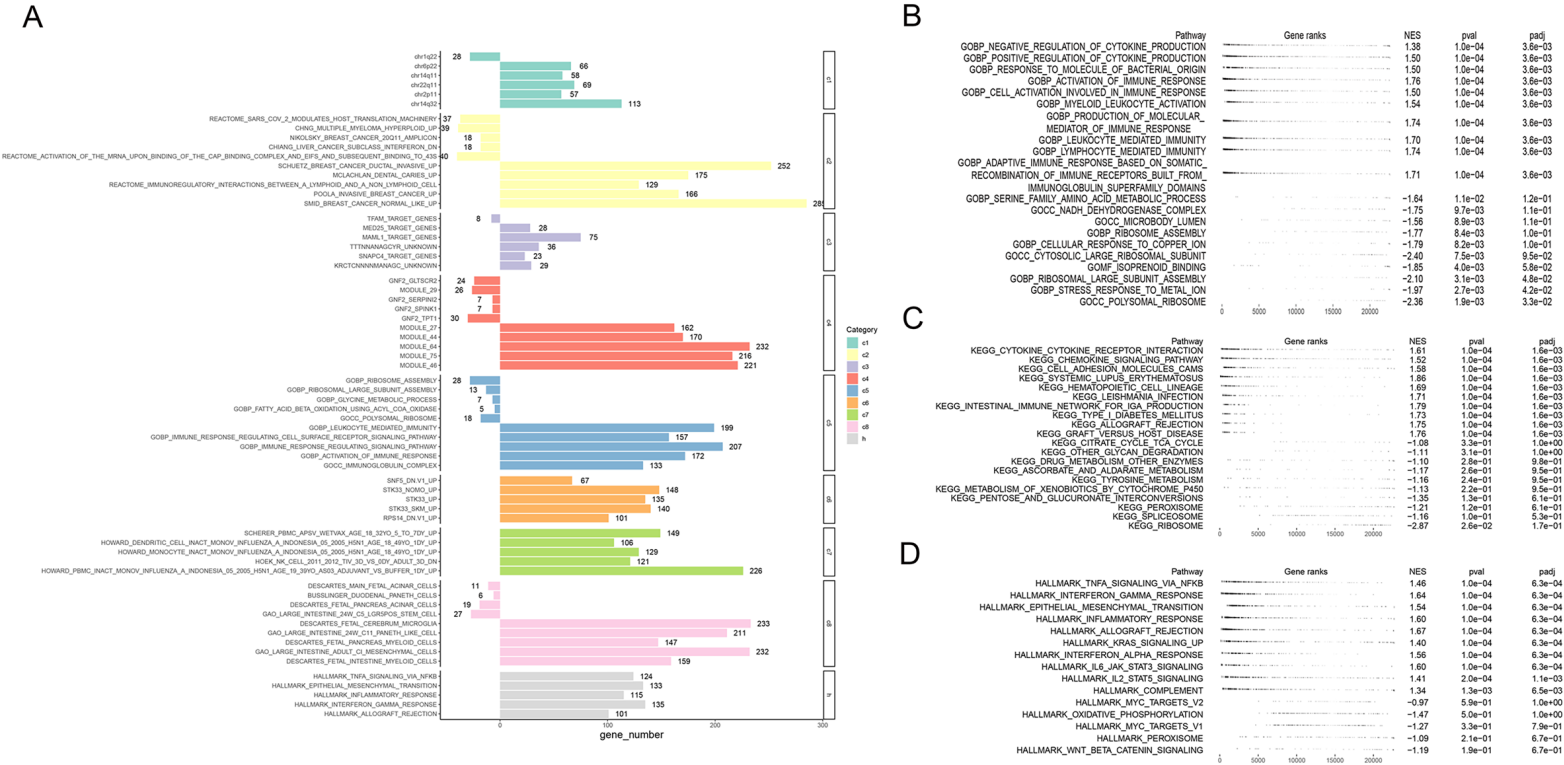
Functional enrichments were performed. (**A**) The result of enrichment analyses based on nine gene sets was shown (independent samples *t* test). (**B**–**D**) The top 20 enriched GO terms, KEGG pathways and 15 hallmark pathways were displayed.

To unearth the potential molecular mechanisms of AIM2, we performed GSEA on 432 COAD patients utilizing nine different gene sets. Figure 6A depicted the enrichment of DEGs within these gene sets. The high expression group showed significant enrichment in the top five GO analysis terms: negative regulation of cytokine production, positive regulation of cytokine production, response to bacterial origin molecules, immune response activation, and cell activation involved in the immune response. In contrast, the low expression group significantly enriched terms related to serine family amino acid metabolic processes, NADH dehydrogenase complex, microbody lumen, ribosome assembly, and cellular response to copper ions (Fig. 6B). The KEGG analysis results showed that these DEGs were enriched in cytokine-cytokine receptor interaction, chemokine signaling pathway, cell adhesion molecules cams, systemic lupus erythematosus, and hematopoietic cell lineage in high expression group. In the low expression group, these DEGs showed a high level of association with the citrate cycle TCA cycle, other glycan degradation, drug metabolism other enzymes, ascorbate, and aldarate metabolism, and tyrosine metabolism (Fig. 6C). In addition, the top five markedly enriched pathways in the high expression group included TNFA signaling via NFKB, interferon-gamma response, epithelial-mesenchymal transition, inflammatory response, and allograft rejection. Low expression groups displayed strong enrichment in five pathways: MYC targets V2, oxidative phosphorylation, peroxisome, and WNT beta-catenin signaling (Fig. 6D).

### A comparison of two expression groups in terms of immunity and immunotherapy

**Figure 7:**
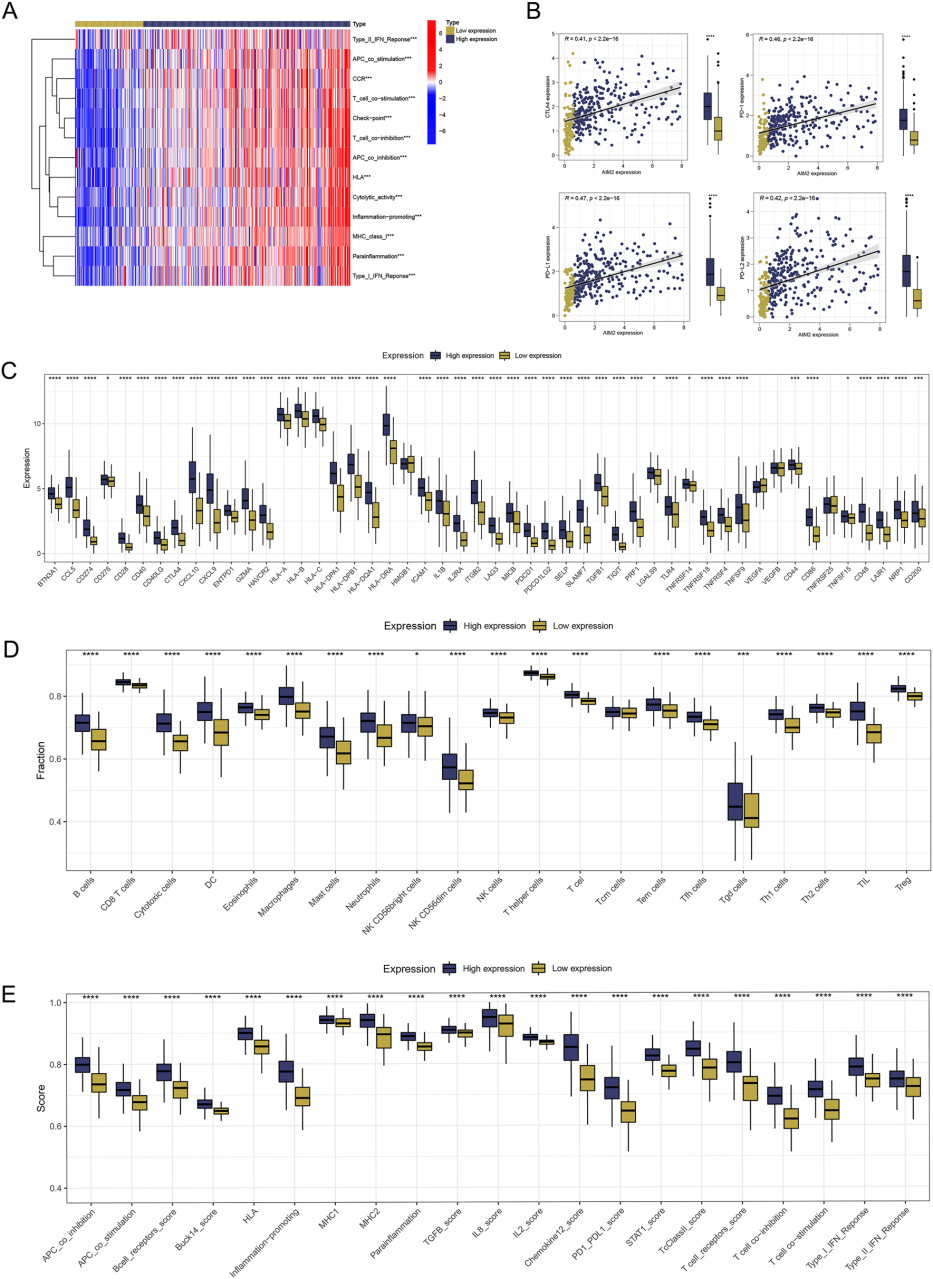
The association between immune infiltration and AIM2 expression was identified. (**A**) The heatmap showed the difference in immune scores between high and low groups, ***P < 0.001 (independent samples *t* test). (**B**) The relationship of AIM2 expression and CTLA4, PD1, PD-L1 and PD-L2 expression were shown, ****P < 0.0001 (Pearson’s correlation coefficient). (**C**) Comparison of immune checkpoint expression between two expression groups, *P < 0.05, ***P < 0.001, ****P < 0.0001 (independent samples *t* test). (**D**,**E**) Comparison of scores of immune cells and immune functions quantified by ssGSEA algorithm between two expression groups, *P < 0.05, ***P < 0.001, ****P < 0.0001 (independent samples *t* test).

**Figure 8:**
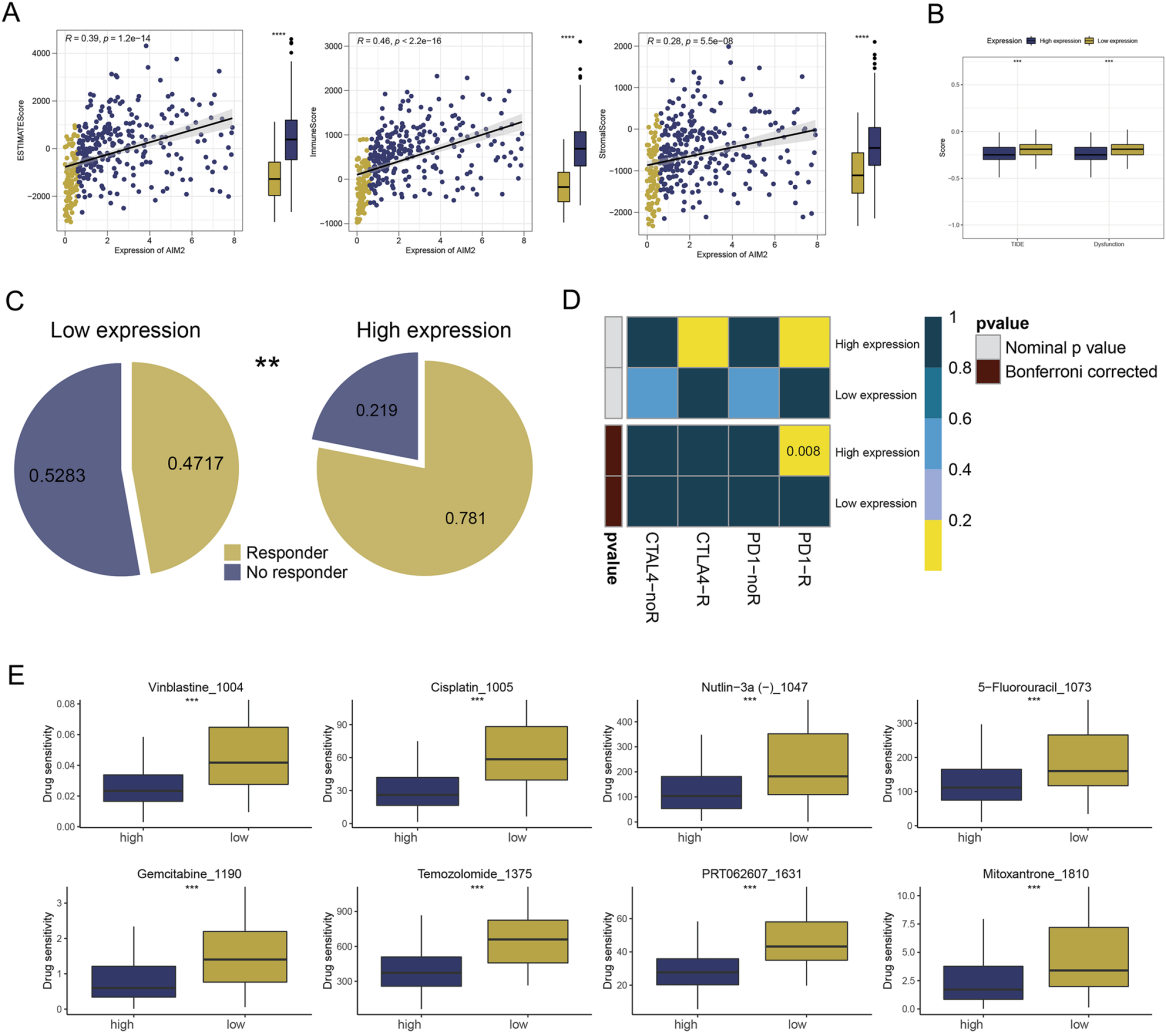
The correlation of immune features and immunotherapeutic response between two expression groups. (**A**) Correlation analysis demonstrated a positive relationship between immune scores, stromal scores, ESTIMATE scores and AIM2 expression, ****P < 0.0001 (Pearson’s correlation coefficient). (**B**) TIDE and dysfunction scores were lower in patients of high expression group than in patients of the low expression group, ***P < 0.001 (independent samples *t* test). (**C**) Patients in high expression group had more responders to immunotherapy compared to low expression group, **P < 0.01 (Chi-squared test). (**D**) Subclass mapping analysis predicted that patients in high expression group were more likely to respond to PD-1 inhibitor therapy (independent samples *t* test). (**E**) Prediction of top eight drugs that may benefit patients, ***P < 0.001 (independent samples *t* test).

Utilizing heatmaps, we highlighted a significant discrepancy between the two groups in terms of the 13 immune-related function scores (Fig. 7A). There was a significant positive correlation between the expression of AIM2 genes and four immune checkpoints. Additionally, patients in the high expression group exhibited higher expression of these immune checkpoints compared to those in the low expression group (Fig. 7B). Intriguingly, patients in the high expression group displayed higher expressions of 50 immune checkpoints than those in the low expression group (Fig. 7C). We analyzed the correlation between AIM2 gene expression and the expression of immune cells and immune-related function scores. Echoing the results of the immune checkpoint analysis, the high expression group had higher immune cell and immune-related function scores than the low expression group. Apart from Tcm cells, the differences were statistically significant (Fig. 7D,E). We calculated immune scores, stroma scores, and ESTIMATE scores, revealing a positive correlation with AIM2 expression (Fig. 8A). In addition, the patients in the high expression group scored higher than low expression group. A TIDE analysis of the COAD cohort revealed that patients with high AIM2 gene expression had lower TIDE and dysfunction scores, indicating a greater likelihood of responding to immunotherapy (Fig. 8B). Patients with low TIDE scores are generally more responsive to immunotherapy than those with high scores^26^. TIDE results suggested that the patients of the high expression group responded better to immunotherapy (Fig. 8C). Subclass mapping analysis was performed to corroborate the TIDE prediction^27^. For patients in the high expression group, anti-PD-1/PD-L1 checkpoint inhibitor therapy was likely to be more beneficial with P < 0.01 (Fig. 8D). Finally, we estimated the IC50s of 179 drugs from the GDSC database using R’oncoPredict and determined drugs with significant sensitivity differences. According to Fig. 8E, the eight drugs with the most significant difference in sensitivity between high and low expression groups were Vinblastine, Cisplatin, Nutlin-3a (–), 5-Fluorouracil, Gemcitabine, Temozolomide, PRT062607, and Mitoxantrone.

### Knockdown of AIM2 promotes colon cancer proliferation, invasion and migration

**Figure 9:**
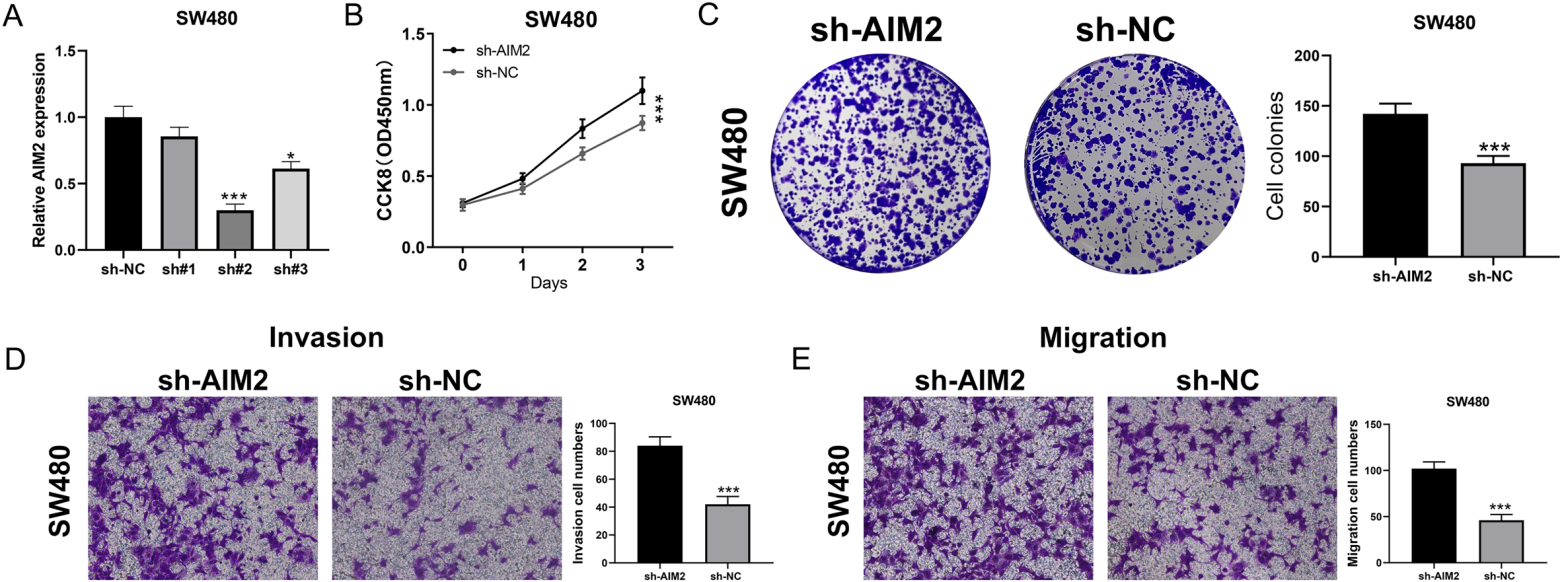
Knockdown of AIM2 promotes colon cancer progression. (**A**) qPCR was used to evaluate the knockdown efficiency of AIM2, *P < 0.05, ***P < 0.001 (Mann–Whitney *U* test). (**B**) CCK8 assay was performed in the sh-NC and sh-AIM2 cells, ***P < 0.001 (Mann–Whitney *U* test). (**C**) Colony formation assay was performed in the sh-NC and sh-AIM2 cells, ***P < 0.001 (Mann–Whitney *U* test). (**D**,**E**) Transwell assay was performed in the sh-NC and sh-AIM2 cells, ***P < 0.001 (Mann–Whitney *U* test).

Subsequently, we sought to investigate the role of AIM2 in colon cancer. The knockdown efficiency of AIM2 was depicted in Fig. 9A. The shRNA#2 has the best efficiency and therefore was selected for further experiments. CCK8 and colony formation assays indicated that the knockdown of AIM2 could significantly promote colon cancer proliferation (Fig. 9B,C). Transwell assay showed that the inhibition of AIM2 could remarkably enhance the invasion and migration ability of colon cancer cells (Fig. 9D,E).

### AIM2 inhibits the growth and metastasis of colon cancer cells in vivo

**Figure 10:**
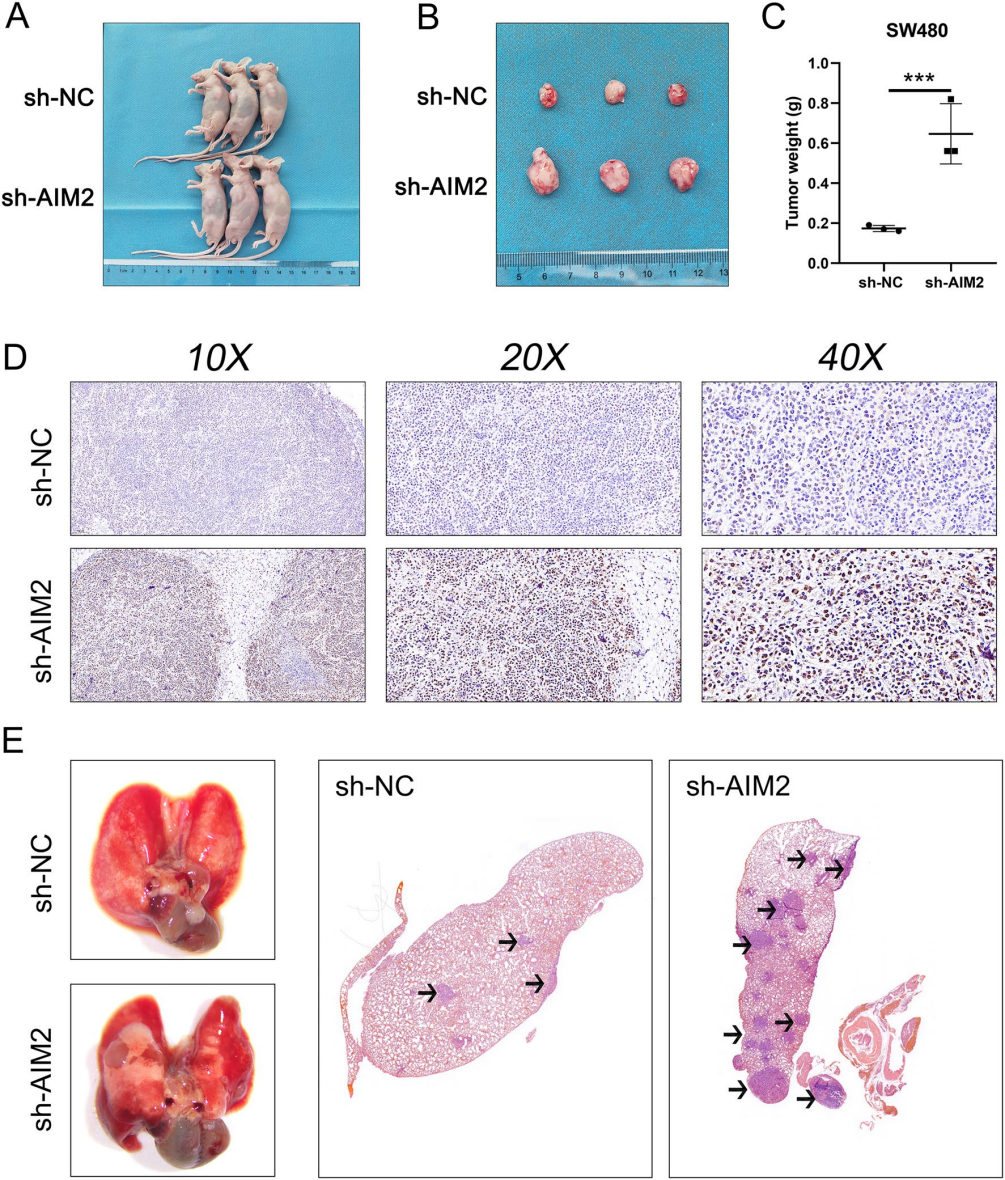
Knockdown of AIM2 promotes the growth and metastasis of colon cancer cells in vivo. (**A**,**B**) Images of xenograft tumors after injection of SW480 cells transfected with sh-NC and sh-AIM2. (**C**) The subcutaneous tumor weights were weighed at the endpoint time of the experiment, ***P < 0.001 (Mann–Whitney *U* test). (**D**) Ki67 staining of subcutaneous tumor. (**E**) Images of lung metastasis model after injection of SW480 cells transfected with sh-NC and sh-AIM2.

In order to substantiate the role of AIM2 in colon cancer, we utilized both the subcutaneous xenograft model and the lung metastasis model. For the xenograft model, we randomly assigned nude mice into two categories. SW480 cells displaying consistent sh-AMI2 expression and a control group (sh-NC) were subcutaneously administered into each mouse. The observed data revealed a significant retardation of tumor growth in the sh-AIM2 group relative to the sh-NC group (Fig. 10A,B). This led to the observation that mice receiving sh-AIM2 injections displayed increased tumor weight at the experiment's conclusion, as compared to the sh-NC group (Fig. 10C). In a subsequent analysis, IHC examination revealed an inverse correlation between Ki67 expression levels and AIM2 in the mice's orthotopic tumor tissues (Fig. 10D). In parallel, the lung metastasis model highlighted that the lung tissue from the sh-AIM2 group had a higher incidence of metastases than the sh-NC group (Fig. 10E). Summarily, our evidence suggests that AIM2 plays a critical role in impeding both the proliferation and metastasis of colon cancer in vivo.

### Knockdown of AIM2 promotes macrophage polarization to the M2 phenotype

**Figure 11:**
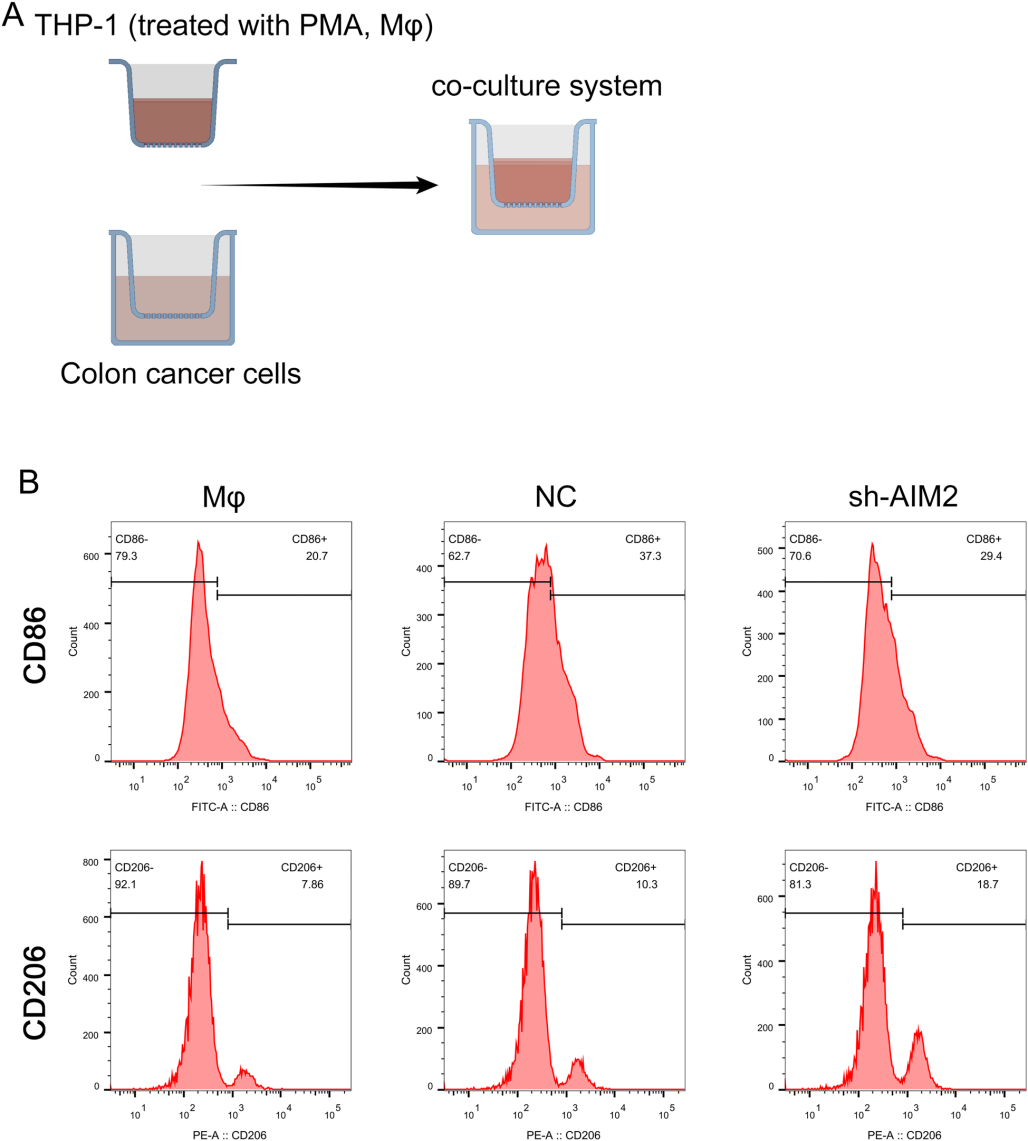
Knockdown of AIM2 promotes macrophage polarization to the M2 phenotype. (**A**) Co-cultivation mode diagram. (**B**) Flow cytometric analysis of the expressions of CD206/CD86 in macrophages co-cultured with colon cancer cells with AIM2 knockdown and control cells. Numerical values denote the relative fluorescence intensity.

We employed the CIBERSORT algorithm to delve deeper into the differential immune infiltration between patients exhibiting high and low AIM2 expression. Our analysis suggested that those with elevated AIM2 expression could have increased infiltration levels of CD8^+^ T cell, memory activated CD4^+^ T cell, activated NK cell, and M1 macrophage, but decreased infiltration of naïve B cell, plasma B cell, resting memory CD4^+^ T cell, resting NK cell, and M2 macrophages (Fig. S1). In order to explore if AIM2 might influence macrophage TAM polarization, we instituted a co-culture system with THP-1 (Mφ) cells (Fig. 11A). Following this, we probed the alterations in M1 and M2 marker expression. As hypothesized, AIM2 silencing led to a decline in the M1 marker CD86, while concurrently enhancing the M2 marker CD206 expression. This outcome implies that AIM2 can influence the TAM polarization process, directing it more towards M1 polarization (Fig. 11B).

## Discussion

Colorectal cancer, with colon adenocarcinoma (COAD) as the most prevalent type, ranks as the third most common cancer and the second leading cause of cancer-related deaths globally^28^. Recent years have witnessed the rise of immunotherapy as a treatment modality for tumors^29^. By inhibiting immune checkpoints, T cells can be mobilized to target tumor cells. In the management of melanoma, non-small cell lung cancer, and bladder cancer, immune checkpoint inhibitors (ICIs) have shown remarkable outcomes^30^. However, the responses to ICIs can vary significantly among patients, and their lower response rates limit their widespread clinical adoption^31^. A deeper understanding of COAD's genetic and epigenetic aspects can aid in identifying new biomarkers and therapeutic targets, thereby enhancing diagnostic precision and treatment efficacy.

ICD is a form of cell death induced by drugs, viruses, radiation, and other agents, leading to T cell immune activation and the release of damage-associated molecular patterns (DAMPs)^32^. Tumor-specific T cells are drawn to tumors by the biomarkers associated with ICD, such as calreticulin, high-mobility group box 1, and ATP^33^. In this process, the T cells recruited to the tumor microenvironment become reactivated to attack the cancer cells. As such, COAD drugs should be assessed for their capacity to induce the release of ICD-associated biomarkers. The combination of these drugs with immunomodulators may yield superior anti-tumor effects compared to surgery, radiation therapy, chemotherapy, or targeted therapy alone.

Analysis of publicly available data can guide researchers in their studies^34–37^. In this study, we calculated the score of each patient’s ICD-related genes using the ssGSEA algorithm and conducted differential expression analysis of 32 ICD-related genes. We identified AIM2 as a specific prognostic gene through Cox analysis and found that AIM2 was overexpressed in tumor samples compared to normal samples. Various studies have established that AIM2 plays a critical role in cancer development and progression, displaying both pro-cancer and oncogenic activities. F For instance, AIM2 knockdown inhibits the proliferation, migration, and enlargement of gastric cancer cells, thereby decelerating tumor progression^38^. Furthermore, AIM2 may inhibit osteosarcoma growth through the inactivation of the PI3K/AKT/mTOR signaling pathway, suggesting its potential as a therapeutic marker^39^. Nonetheless, our understanding of AIM2’s role in COAD remains limited. Research has also indicated that DNA damage can influence both innate and adaptive immune responses through nucleic acid-sensing mechanisms. Targeted DNA repair therapies depend on these proteins for their effectiveness, with AIM2 playing a pivotal role in this process^40^. These findings suggest that further examination of AIM2 could yield clinically valuable insights for COAD treatment.

Neoantigen load, also referred to as tumor mutational burden (TMB), quantifies the number of non-silent somatic coding mutations present within a tumor's coding region. This measure can indicate the effectiveness of ICIs in clinical practice^41^. In our analysis, we found that patients with high AIM2 expression had elevated TMB levels, suggesting a potential increased responsiveness to immunotherapy. Stemness, referring to a cell’s potential for self-renewal and differentiation, is often associated with cancer stem cells (CSCs). CSCs, possessing traits similar to normal stem cells, are believed to sustain and proliferate tumors^42^. In this study, we calculated the stemness index (mRNAsi) and the other index (mDNAsi) of each COAD patient using one-class logistic regression (OCLR) algorithm^14^, and determined that patients with high AIM2 expression demonstrated increased mRNAsi scores. Moreover, our copy number variation (CNV) analysis showed a lower burden of copy number gain or loss in patients with high AIM2 expression. These findings suggest that COAD patients with high AIM2 expression may exhibit enhanced responses to immunotherapy.

The tumor microenvironment (TME), comprising various cells, plays a crucial role in tumor progression. Numerous studies have explored the influence of immune cells in tumor tissues in initiating and advancing tumors. We noticed that those with elevated AIM2 expression could have increased infiltration levels of CD8^+^ T cell, memory activated CD4^+^ T cell, activated NK cell, and M1 macrophage. Activated NK cells, CD8^+^ T cells, and M1 macrophages are integral to cancer prevention and treatment strategies. NK cells, integral to the body’s innate immune response, can identify and annihilate their own cells that have undergone malignant transformations, thereby thwarting the onset of tumors^43^. CD8^+^ T cells, also termed killer T cells, can specifically recognize and exterminate cancer cells presenting specific antigens, hence curtailing cancer proliferation^44^. M1 macrophages, a category of immune cells, possess potent inflammatory responses and can secrete a wealth of inflammatory factors like TNF-α and IL-12^45^. These cells can also phagocytize cancer cells, thus impeding cancer progression. The positive correlation of AIM2 to these cells might partly explain the anticancer effect of AIM2 in colon cancer. Our study results showed considerable differences in immune cell abundance, immune function scores, and immune checkpoint expression between the two groups. It's plausible that AIM2 plays a significant role in tumor immunity. CTLA4, PD-1, PD-L1, and PD-L2 expression was significantly higher in the high expression group. These patients also scored higher on immune cell and immune function evaluations compared to the low expression group. Therefore, immunotherapy could be advantageous for patients in the high expression group, potentially boosting their immune reactivity and improving their prognosis. We further verified this hypothesis using the TIDE algorithm. The high expression group exhibited lower TIDE scores, indicating successful immunotherapy. Subclass mapping algorithms confirmed this notion, revealing a favorable response to anti-PD-1 therapy in patients within the high expression group. In our research, AIM2 expression effectively predicts patients' responses to immunotherapy.

It's important to note that our study has several limitations. Firstly, our cohort size was relatively small, and all samples were collected from Western countries. Secondly, metabolomics and proteomics analyses could provide insight into the molecular mechanisms behind COAD immunobiology, beyond what can be gleaned from transcriptomics analysis alone. Thirdly, additional experiments are required to confirm the prognostic and immunotherapeutic roles of AIM2 in COAD. Lastly, the functional modalities of genes are often multifaceted and complex, and our analysis might have only partially revealed the various roles of AIM2 in the context of cancer. Given this, our findings provide limited insight into the intricate mechanistic functions of AIM2. High-quality studies exploring the broader and deeper roles of AIM2 in greater detail are thus imperative for a more comprehensive understanding.

### Supplementary Information

Supplementary Information.

Supplementary Figure S1.

## Data Availability

All the data used in our study are included in the article and further inquiries can be directed to the corresponding author.
