# Supplementary material for: Prognostic and immunotherapeutic significance of immunogenic cell death-related genes in colon adenocarcinoma patients
Source: Sci Rep. 2023 Nov 6;13:19188. doi: 10.1038/s41598-023-46675-y (PMC10628212; doi:10.1038/s41598-023-46675-y)
Supplement: Supplementary file 1 — Supplementary Information. [file 41598_2023_46675_MOESM1_ESM.docx]

**Figure legends**

**Figure S1. The immune microenvironment quantified by CIBERSORT algorithm in colon cancer patients with high and low AIM2 expression**

**Notes:** * = P < 0.05, ** = P < 0.01, *** = P < 0.001 (Independent Samples t-test)
