# Supplementary figures and images for: Prognostic and immunotherapeutic significance of immunogenic cell death-related genes in colon adenocarcinoma patients
Source: Sci Rep. 2023 Nov 6;13:19188. doi: 10.1038/s41598-023-46675-y (PMC10628212; doi:10.1038/s41598-023-46675-y)

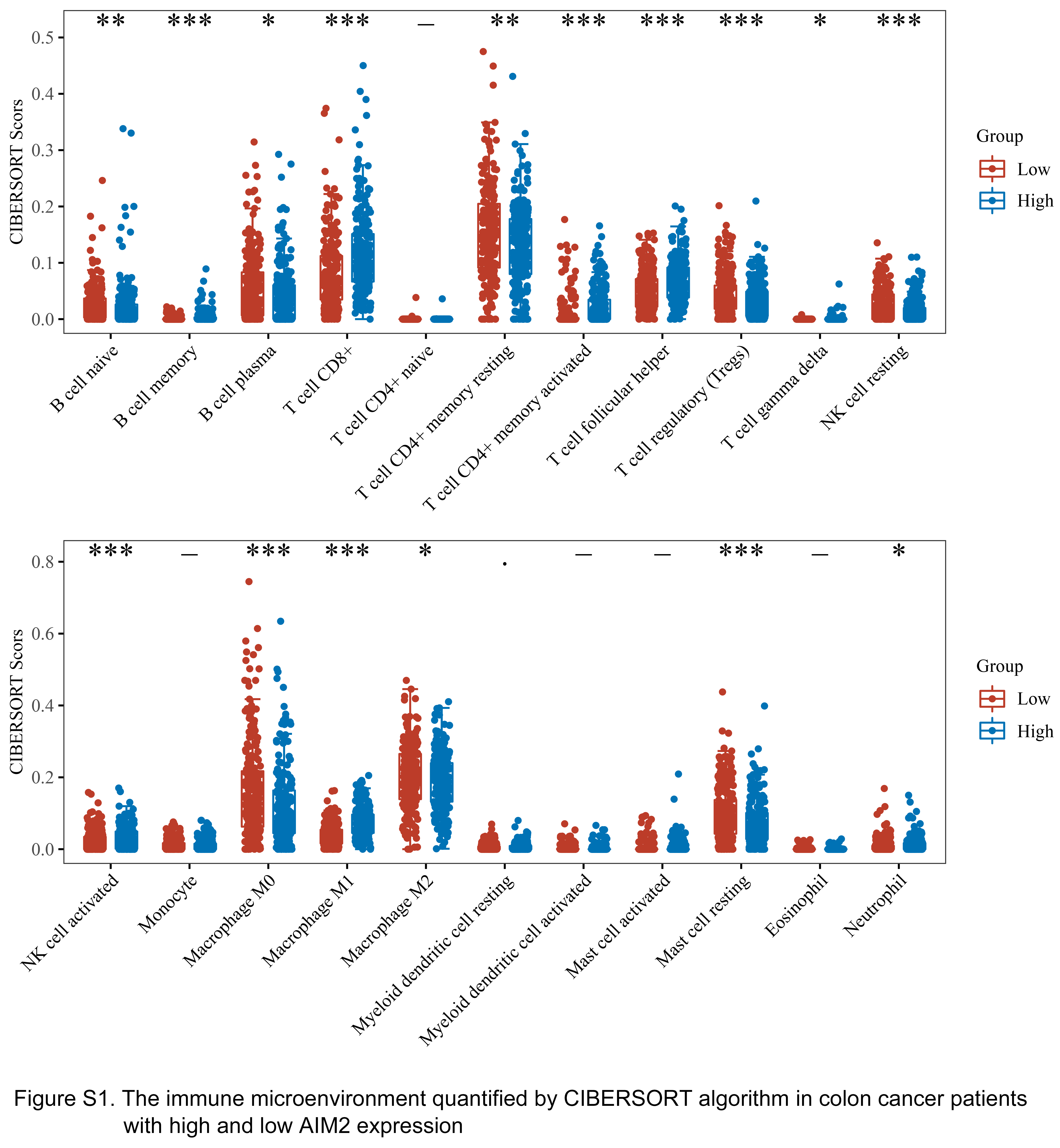

Supplement: Supplementary file 2 — Supplementary Figure S1. [file 41598_2023_46675_MOESM2_ESM.tif]
